# Theoretical Study on the Multiple Free Radical Scavenging Reactions of Pyranoanthocyanins

**DOI:** 10.3390/antiox13010033

**Published:** 2023-12-22

**Authors:** Yapeng Du, Yu Chai, Xiaoping Zheng, Yanzhen Zheng

**Affiliations:** College of Ocean Food and Biological Engineering, Jimei University, Xiamen 361021, China; dfcfafu126@126.com (Y.D.); fzmfafu126@126.com (Y.C.); grfafu126@126.com (X.Z.)

**Keywords:** pyranoanthocyanins, flavonoid derivatives, antioxidative activity, density functional theory, mechanism

## Abstract

The free radical trapping capacities of multiple pyranoanthocyanins in wine storage and ageing were theoretically explored by density functional theory (DFT) methods. Intramolecular hydrogen bonds were detected in all pyranoanthocyanins, and the planarity of the compounds worsened with an increasing dielectric constant in the environment. Solvents significantly influenced the reaction enthalpies; thus, the preferred thermodynamic mechanisms of the free radical scavenging reactions were modified in different phases. This study incorporates hydrogen atom transfer (HAT), proton loss (PL), electron transfer (ET) reactions, and demethylation (De) of methoxy group mechanisms. The three pyranoanthocyanins have the capacity to capture n_1_+1 free radicals, where n_1_ represents the number of methoxy groups. In the gas phase, they prefer employing the n_1_-De-HAT mechanism on the guaiacyl moiety of the B ring, resulting in the formation of a stable quinone or a quinone radical to scavenge free radicals. In the benzene phase, pyranoanthocyanins trap free radicals via a PL−n_1_−De−HAT mechanism. In the water phase, the targeted pyranoanthocyanins may dissociate in the form of carboxylate and tend to utilize the n_2_−PL−n_1_−De−ET mechanism, where n_2_ and n_1_ represent the number of phenolic groups and methoxy groups, respectively, facilitating multiple H^+^/e^−^ reactions.

## 1. Introduction

Anthocyanins, a class of flavonoid compounds, are natural pigments widely found in plants [1,2,3,4]. The chemical reactions of anthocyanins are closely related to the color changes in the wine during storage and ageing [5]. Anthocyanins are highly reactive and can react with other constituents (polyphenols, aldehydes, etc.) in red wine, yielding different anthocyanin derivatives [6,7]. In reactions with low-molecular-weight molecules such as acetoacetic acid, acetaldehyde, pyruvic acid, vinylguaiacol, vinylcatechol, vinylphenol, and vinylcatechin, anthocyanins generate an important class of derivatives called pyranoanthocyanins. These compounds are more stable at larger pH ranges than anthocyanin monoglucosides. Their spectroscopic features are considered to present a more orange−red color exhibited during wine maturity and ageing [6,7].

Free radicals produced in the human body are considered to be a vital element for ageing and different kinds of chronic diseases, such as cardiovascular diseases, neurological disorders and diabetes [8,9,10]. Hence, searching for antioxidants to eliminate the free radicals is important for health [9,10]. Pyranoanthocyanins demonstrate some antioxidant characteristics in the same way as anthocyanin precursors. Their special structure permits easy hydrogen atom/electron transfer and can improve activity to delocalize unpaired electrons through conjugation in the aromatic ring [11]. Therefore, pyranoanthocyanins are regarded as good traps for free radicals [12].

The free radical-capturing properties are estimated by antioxidant activities. Specifically, the development of computational methodologies enhances the opportunities for theoretical methods to be widely used in estimating the free radical-capturing property of a compound [13,14,15,16,17,18,19,20,21]. Theoretical methods, especially DFT calculations, with an equivalent or even better accuracy than the experiments, can reveal the molecular intrinsic mechanism for the free radical scavenging properties and disclose the relationship of the structure–antioxidant properties [13,14,15,16,17,18,19,20,21]. They can help to understand the free radical-capturing mechanisms of different molecules, clarify controversial experimental facts, complement the experimental findings, or even help to provide effective information for the designing excellent antioxidants. Combined with the experimental and theoretical calculations, the molecular basis arising from the free radical trapping reaction for the antioxidant capacities of polyphenol compounds, including anthocyanins, is suggested: hydrogen atom transfer and electron transfer.

The antioxidant properties of pyranoanthocyanins have been studied before [22,23,24]. Previous studies have found that pyranoanthocyanins, bearing the catechol moiety or the syringyl unit, are effective antioxidants to trap free radicals by inactivating them [22]. With a higher degree of electron delocalization, the single-electron transfer mechanism is more possible [22]. The catechol or o-dimethoxy motif also increases the antioxidant capacity, which makes pyranoanthocyanins better antioxidants than the malvidin-3-glucoside [22].

**Figure 1:**
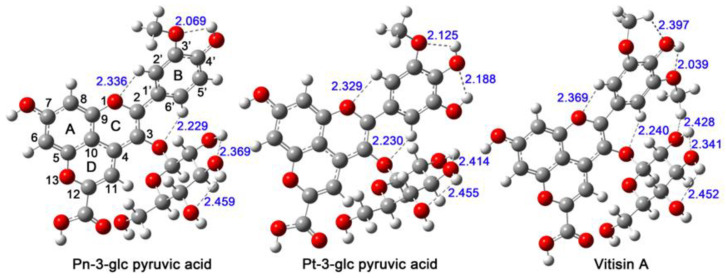
The most stable structures of the investigated compounds. The atom numberings for the studied compounds are labeled in Pn-3-glc pyruvic acid. The hydrogen bonds are marked by dashed lines, and the corresponding H···O distances are labelled in the optimized geometries.

Polyphenolic molecules containing catechol/guaiacyl motifs can undergo more than one H^+^/e^−^ reaction to trap multiple free radicals [25,26,27,28]. Pyranoanthocyanins have catechol moieties or guaiacyl motifs. However, previous works related to the molecular basis of the antioxidant process were concerned with their single H^+^/e^−^ reaction and the antioxidant capacities of the aromatic OH groups [22,23,24]. For their multiple H^+^/e^−^ processes, more work needs to be performed. Regarding the usefulness of the quantum chemical calculations and as an important section of the continued study about the free radical scavenging capacities of polyphenolic compounds, DFT methods were used to effectively excavate the H^+^/e^−^ processes of pyranoanthocyanins in this work. Three naturally occurring pyranoanthocyanins (Pt-3-glc pyruvic acid, Pn-3-glc pyruvic acid and Vitisin A) containing a catechol motif/guaiacyl unit on the B ring and a carboxyl group on the D ring (Figure 1) were selected. The reaction enthalpies involved in various antioxidant mechanisms were calculated in different solvents. Three environments were considered: the water phase is computed as almost all physiological liquids can find water. Another possible place for the scavenging reaction is the biological membrane (unsaturated lipid); and in this case, the benzene (nonpolar solvent) phase has been considered. For a better comparison, the consideration of a gas environment was also taken into account. Through these systematic investigations, the precise mechanism underlying multiple H^+^/e^−^ processes of pyranoanthocyanins may be unclear.

## 2. Computational Section

The Gaussian 16 software package was applied in the calculations [29]. Gaussview 6 [30] was applied to generate the initial structures, visualize the optimized configurations of the compounds under investigation and analyze the optimized results. In this study, we conducted optimizations on multiple structures of the investigated pyranoanthocyanins using diverse initial conformations. Taking Pn-3-glc pyruvic acid as an example, various initial structures were proposed during the optimization process to achieve the optimized geometries. Several stable structures were obtained; and in the manuscript, we have presented the most stable geometry characterized by the lowest interaction energy among these structures. Geometry optimizations were first performed using the M06-2X/6-31G(d) level in the gas phase [31,32]. Then, the optimized geometries were reoptimized in the solvent phases. All the most stable geometries were confirmed with no imaginary frequencies being real minima. Finally, the single point energies of different species were obtained on the M06-2X/6-311+G** level [32,33]. The solvent effects were applied the SMD continuum solvent model [34].

All the possible species in the antioxidant process, including parent pyranoanthocyanins and all the intermediates (radicals, anions, radical cations, radical anions, and benzodioxoles), can conduct hydrogen atom transfer (HAT), proton loss (PL), and electron transfer (ET) reactions [13,17,19]. For the compound bearing methoxy groups, demethylation (De) of the OMe group may also occur [35,36,37]. The enthalpies related to these reactions were all computed, and the related formulas are listed below (applying the parent pyranoanthocyanins (AH) as the example):

HAT: bond dissociation enthalpy (BDE)

BDE= *H*(A•) + *H*(B•) − *H*(AB) (1)

*H*(A•)—enthalpy of radical (A•); *H*(B•)—enthalpy of hydrogen atom (H•) or methyl radical (•CH_3_); *H*(AB)—enthalpy of AB.

ET: ionization potential (IP) is applied to characterize the enthalpy related to the ET process from the parent molecule.

IP = H(AH•^+^) + *H*(e^−^) − *H*(AH) (2)

*H*(AH•^+^)—enthalpy of radical cation (AH•^+^); *H*(e^−^)—enthalpy of electron (e^−^). 

Electron transfer enthalpy (ETE) was used to characterize the enthalpy related to the other ET process.

ETE= *H*(Ar•) + *H*(e^−^) − *H*(Ar^−^) (3)

*H*(A^−^)—enthalpy of anion (A^−^). PL: proton affinity

(PA) = *H*(A^−^) + *H*(H^+^) − *H*(AH) (4)

*H*(H^+^)—enthalpy of proton (H^+^).

The enthalpies of e^−^, H^+^ and H• were obtained directly on the previous research [38,39,40].

## 3. Results

Three pyranoanthocyanins (Figure 1) containing a carboxyl group at C12 were chosen for the investigation. The atom numberings of the three pyranoanthocyanins are marked in Pn-3-glc pyruvic acid. The other atoms applied the numbers of the attached carbon atoms. For instance, atoms on the C7 atom of Pn-3-glc pyruvic acid are O7 and H7.

### 3.1. The Most Stable Structures of the Pyranoanthocyanins

The geometrical characteristics of a compound are significantly influenced by the environment. The selected geometrical properties in the different phases are given in Table 1. We should stress that pyranoanthocyanins may dissociate in the form of carboxylate anions in the water phase and pyranoanthocyanin carboxylates were considered. The dihedral angles of O1–C2–C3–C4/C10–C9–O1–C2, C10–C5–O13–C12/C10–C4–C11–C12 and C6′–C1′–C2–C3/C6′–C1′–C2–O1 characterize the planar features of the chromone part, the pyruvic acid part with the chromone part and the pyranoanthocyanins, respectively. In Table 1, the dihedral angles of O1–C2–C3–C4 and C10–C9–O1–C2 are approximately 180°. Similar results can be noted for C10–C5–O13–C12/C10–C4–C11–C12. Thus, the results state that the chromone part of the three pyranoanthocyanins and the pyruvic acid part with the chromone part are almost in a plane in the studied phases. Thus, the A, C and D rings of the investigated compounds are almost in a plane. The dihedral angles of C6′–C1′–C2–C3 and C6′–C1′–C2–O1 are in the range of 20.5~32.5° and –147.8~–159.9°, respectively. These data deviate from 180°, which indicates that the phenyl substituent (B ring) and chromone part are not on a plane. Thus, the three pyranoanthocyanins are nonplanar. Moreover, as seen in the table, from the gas phase to the benzene phase and the water phase, the relative dihedral angles of C6′–C1′–C2–C3 and C6′–C1′–C2–O1 increasingly deviated from 180°, which indicates that the planarity of pyranoanthocyanins worsened.

In Figure 1, the distances between H2′ and O1, H4′ and O3′, H6′ and O3 in all three pyranoanthocyanins and those between H5′ and O4′ in Vitisin A and Pt-3-glc pyruvic acid are in the range of 2.069~2.459 Å. They are smaller than 2.5 Å (the sum of van der Waals atomic radii of hydrogen and oxygen), which means that intramolecular hydrogen-bond interactions exist in the studied pyranoanthocyanins [41].

### 3.2. The Favored Mechanism in the Gas Phase

**Figure 2:**
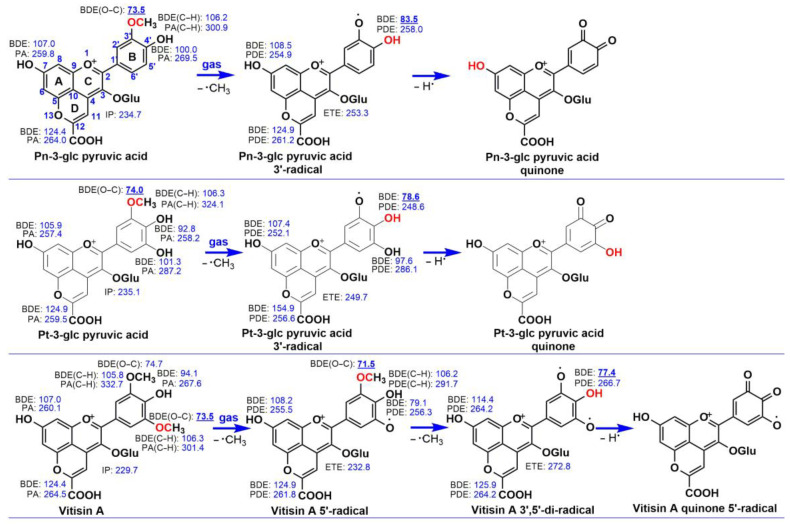
The preferred mechanisms proposed for the radical scavenging reaction of the investigated compounds in the gas phase. The assignment of a positive charge and radical centers in different species is only formal; the charge and unpaired electron are delocalized over the pyranoanthocyanin skeleton. The values for BDE, PA/PDE and IP/ETE are in kcal/mol. The lowest reaction enthalpy in each species is in bold and underlined. The most likely reaction site is in red. The BDE of the C−O bond and BDE/PA/PDE of C−H bond have been denoted as BDE(O−C) and BDE/PA/PDE(C−H), respectively. Those of the other BDEs, PAs and PDEs are from the phenolic OHs.

**Figure 3:**
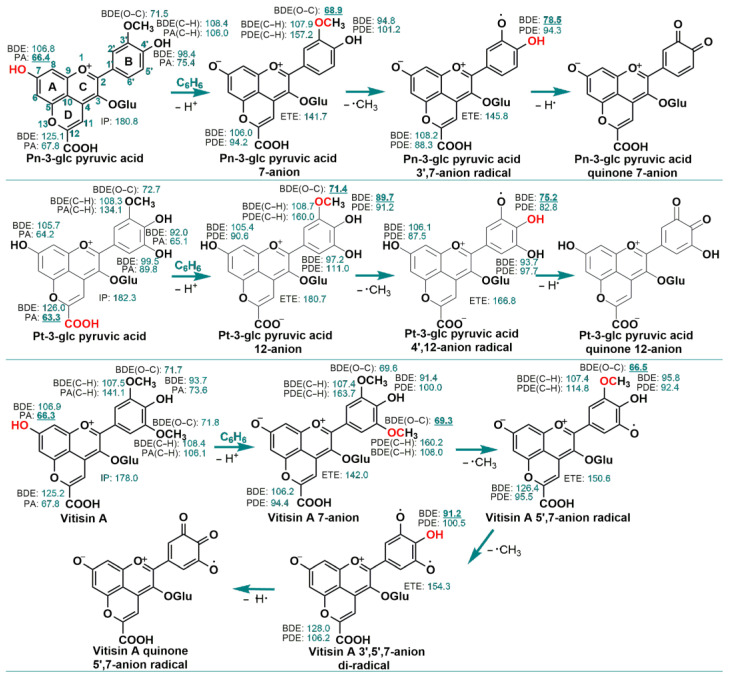
The preferred mechanisms proposed for the radical scavenging reaction of the investigated compounds in the benzene phase. The assignment of a positive charge, a negative charge and radical centers in different species is only formal; the charge and unpaired electron are delocalized over the pyranoanthocyanin skeleton. The values for BDE, PA/PDE and IP/ETE are in kcal/mol. The lowest reaction enthalpy in each species is in bold and underlined. The most likely reaction site is in red. The BDE of the C−O bond and BDE/PA/PDE of C−H bond have been denoted as BDE(O−C) and BDE/PA/PDE(C−H), respectively. Those of the other BDEs, Pas and PDEs are from the phenolic Ohs.

**Figure 4:**
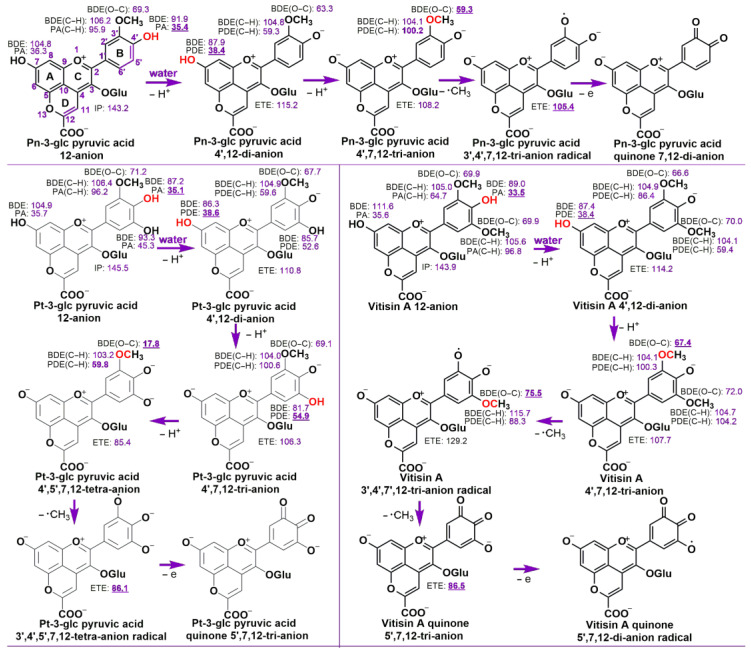
The preferred mechanisms proposed for the radical scavenging reaction of the investigated compounds in the water phase. The assignment of a positive charge, a negative charge and radical centers in different species is only formal; the charge and unpaired electron are delocalized over the pyranoanthocyanin skeleton. The values for BDE, PA/PDE and IP/ETE are in kcal/mol. The lowest reaction enthalpy in each species is in bold and underlined. The most likely reaction site is in red. The BDE of the C−O bond and BDE/PA/PDE of C−H bond have been denoted as BDE(O−C) and BDE/PA/PDE(C−H), respectively. Those of the other BDEs, PAs and PDEs are from the phenolic OHs.

In this study, the possible mechanisms for the multiple free radical-capturing process with the thermodynamic tendency were mainly based on our results. The species may undergo proton loss, electron transfer or hydrogen atom donation in the free radical scavenging reaction. The proton loss, electron transfer and hydrogen atom donation process were all considered in the investigated environments. And the reaction enthalpies are presented in Figure 2, Figure 3 and Figure 4.

Figure 2 illustrates the reaction enthalpies of proton loss, demethylation of the methoxy group, electron transfer, and hydrogen atom donation processes for the three pyranoanthocyanins in the gas phase. Upon comparing the calculated reaction enthalpies of the investigated pyranoanthocyanins, we observed that the reaction energies of C–O bond breaking of the methoxy group are the lowest among the gas-phase reaction enthalpies. For instance, in Figure 2, the BDE(O−C) of Pn-3-glc pyruvic acid is 73.5 kcal/mol, while the IP, the smallest PA (7−OH PA), and the smallest BDE (4′−OH BDE) are 234.7 kcal/mol, 259.8 kcal/mol, and 100.0 kcal/mol, respectively. Similar results are evident for Pt-3-glc pyruvic acid and Vitisin A. Consequently, the investigated pyranoanthocyanins preferentially trap the first free radical via the breaking of the C–O bond of the methoxy group in the gas phase. The Vitisin A radical would continuously lose the methyl radical from the methoxy group due to the lower energy required for C–O bond breaking. After all the methyl groups in the methoxy group are released, the pyranoanthocyanin radicals would lose hydrogen atoms from the hydroxyl group. As depicted in Figure 2, the reaction enthalpies of hydrogen atom transfer from the pyranoanthocyanin radicals are the lowest among the gas-phase reaction enthalpies. For example, in Figure 2, the highest value of BDE for the Pt-3-glc pyruvic acid radical is 154.9 kcal/mol (12−OH BDE), while the ETE and the smallest PDE (4′−OH PDE) are 249.7 kcal/mol and 248.6 kcal/mol, respectively. Similar results are observed for Pn-3-glc pyruvic acid and Vitisin A species. Thus, the second/third free radical scavenging process for the investigated compounds occurs via the HAT mechanism from the OH groups.

Similar to other flavonoids, the functional groups in the B ring play a crucial role in the radical-capturing reactions of parent pyranoanthocyanins in the gas phase. For instance, in Vitisin A, the lowest bond dissociation energy (BDE) is found in 5′–OCH_3_, measuring 73.5 kcal/mol. Comparatively, the BDEs for 7–OH (A ring) and 12–COOH (D ring) are 107.0 kcal/mol and 124.4 kcal/mol, respectively. All these values surpass the BDE of 5′–OCH_3_. Therefore, the three pyranoanthocyanins exhibit a preference for the initial free radical-capturing reaction via the demethylation mechanism on the 5′–OCH_3_ group in the gas phase. Due to the formation of a stable quinone, pyranoanthocyanins then undergo the second/third free radical-capturing reactions at the adjoining 4′−OH group, rather than the other antioxidant groups. We also observed that the production of quinones enhances the antioxidant ability of 4′−OH. For example, the 4′−OH BDE of Pn-3-glc pyruvic acid is 100.0 kcal/mol, exceeding the 4′−OH BDE of the Pn-3-glc pyruvic acid 3′-radical (83.5 kcal/mol). This indicates that the production of quinone can also strengthen the ability of pyranoanthocyanins for other free radical-trapping reactions.

Compared to the previous study [22,23], our work exhibits a similar trend in the BDE value for the hydroxyl group. The 4′-OH group shows the lowest energy required for the loss of the hydrogen atom from the phenolic OH, whereas the 7-OH group demonstrates the highest energies. This outcome further validates the precision of our research.

In general, all three pyranoanthocyanins exhibit a preference for trapping free radicals through the demethylation of the methoxy groups, followed by the HAT mechanism from the 4′−OH groups in the B ring. Pn-3-glc pyruvic acid and Pt-3-glc pyruvic acid scavenge two free radicals in the gas phase through a combination of demethylation and HAT mechanism on the guaiacyl moiety, initially from the 3′–OCH_3_ groups, followed by the 4′–OH. On the other hand, Vitisin A captures three radicals on the guaiacyl moiety, starting with the 5′–OCH_3_ groups, followed by the 3′–OCH_3_ and 4′–OH. Consequently, in the gas phase, the three pyranoanthocyanins can trap n_1_+1 free radicals via the n_1_−De−HAT mechanism, where n_1_ represents the number of methoxy groups.

### 3.3. The Favored Mechanism in the Benzene Phase

Figure 3 illustrates the BDE, IP/ETE, and PA/PDE for the three pyranoanthocyanins and related species in the benzene phase. In the parent Pn-3-glc pyruvic acid, the lowest reaction enthalpy is the 7–OH PA at 66.4 kcal/mol, making the losing proton from the 7–OH being the initial step in the free radical trapping process. The resulting 7−anion then undergoes demethylation with the lowest reaction enthalpy occurring at the 3′–OCH3. Subsequently, the 3′,7−anion radical sequentially loses a hydrogen atom from 4′−OH in the guaiacyl moiety, ultimately forming the quinone 7−anion.

In contrast to Pn-3-glc pyruvic acid, the lowest reaction enthalpy for the parent Pt-3-glc pyruvic acid is the 12–COOH PA at 63.3 kcal/mol. As a result, Pt-3-glc pyruvic acid initially loses a proton from the carboxylic group in the free radical trapping process. The resulting 7−anion continuously loses a methyl group and a hydrogen atom from the guaiacyl moiety, leading to the formation of the quinone 12−anion.

For the parent Vitisin A, the 7−OH PA at 66.3 kcal/mol represents the lowest reaction enthalpy. Therefore, in the free radical trapping process, Vitisin A first loses a proton from the 7–OH. The resulting 7−anion then undergoes sequential loss of two methyl groups and a hydrogen atom from the guaiacyl moiety, resulting in the formation of the quinone 5′,7-anion radical.

In the benzene phase, it is also observed that the generation of quinones enhances the antioxidant ability of 4′−OH. For instance, the 4′−OH BDE of Pt-3-glc pyruvic acid is 92.0 kcal/mol, significantly higher than the 4′−OH BDE of Pt-3-glc pyruvic acid 4′,12-anion radical (75.2 kcal/mol). Thus, the production of quinone also strengthens the ability of pyranoanthocyanins for the second HAT reaction.

In general, all three pyranoanthocyanins would loss one proton in the hydroxyl group in A ring or carboxylic group in the D ring. The pyranoanthocyanin anions exhibit a preference for trapping free radicals through the demethylation of the methoxy groups, followed by the HAT mechanism from the 4′−OH groups in the B ring. Pn-3-glc pyruvic acid anion and Pt-3-glc pyruvic acid anion scavenge two free radicals in the benzene phase through a combination of demethylation and HAT mechanism on the guaiacyl moiety, initially from the 3′–OCH_3_ groups, followed by the 4′–OH. On the other hand, Vitisin A anion captures three radicals on the guaiacyl moiety, starting with the 5′–OCH_3_ groups, followed by the 3′–OCH_3_ and 4′–OH. Consequently, in the benzene phase, the three pyranoanthocyanins can trap n_1_+1 free radicals via the PL−n_1_−De−HAT mechanism, where n_1_ represents the number of methoxy groups.

### 3.4. The Favored Mechanism in the Water Phase

The carboxyl group may dissociate the proton in the water phase and the three pyranoanthocyanins are in the form of carboxylate anions. COOH group deprotonation can affect the studied reaction enthalpies. The calculated reaction energies for different mechanisms of pyranoanthocyanin carboxylates in the water phase are presented in Figure 4. Due to the smaller PL enthalpies for hydroxyl groups, the investigated pyranoanthocyanin carboxylates would prefer to undergo successive proton loss on the hydroxyl groups first followed by methyl group in guaiacyl moiety with the formation of 3′,4′,7,12−tetra−anion for Vitisin A and Pn-3-glc pyruvic acid, and 3′,4′,5′,7,12−penta−anion for Pt-3-glc pyruvic acid. All the 4′−OH PAs were smaller than the other proton transfer reaction enthalpies. For example, in Pn-3-glc pyruvic acid carboxylate, 4′−OH PA is 35.4 kcal/mol, smaller than that of 7−OH PA (36.3 kcal/mol) and 3′−CH PA (95.9 kcal/mol). Therefore, in the water phase, the three pyranoanthocyanin carboxylates favor performing the first proton loss reaction on 4′−OH group. In Figure 4, it is evident that the 4′,12−di−anions exhibit a preference for sequentially releasing proton atoms from the aromatic OH groups in the B ring, following the remaining OH groups in C ring. This results in the formation of 4′,7,12−tri−anions for Vitisin A and Pn-3-glc pyruvic acid, and a 4′,5′,7,12−tetra−anion for Pt-3-glc pyruvic acid. The tri/tetra−anions then proceed to eliminate all methyl groups on the guaiacyl unit following with a one-electron denaturation process. This leads to the formation of a quinone 7,12−di−anion for Pn-3-glc pyruvic acid, a quinone 5′,7,12−tri−anion for Pt-3-glc pyruvic acid, and a quinone 5′,7,12−di−anion radical for Vitisin A.

Overall, in the water phase, all examined pyranoanthocyanin carboxylates initially undergo the loss of all phenolic OH groups, resulting in the formation of tri−anions and tetra−anions. Pn-3-glc pyruvic acid and its tri-anions capture two free radicals through demethylation of the methoxy groups and one electron loss. Vitisin A exhibits an affinity for capturing three free radicals through a successive demethylation process followed by one electron transfer. Consequently, in the water phase, the three pyranoanthocyanin 12-anions can trap n_1_+1 free radicals via the n_2_−PL−n_1_−De−ET mechanism, where n_2_ and n_1_ represent the number of phenolic groups and methoxy groups, respectively.

## 4. Discussion

As depicted in Figure 2, in the gas phase, pyranoanthocyanins exhibit a preference for transferring all methyl groups from the methoxy groups. Subsequently, they tend to abstract the hydrogen atom from the 4′–OH, located in the guaiacyl moiety, akin to the methoxy groups. This leads to the generation of a stable quinone for Pn-3-glc pyruvic acid and Pt-3-glc pyruvic acid, and a quinone radical for Vitisin A. Similar outcomes are observed in the benzene phase due to the lower energy required for the dissociation of the 4′−OH bond compared to other reaction enthalpies involving the pyranoanthocyanins 3′−radical and 3′,5′−di−radical. In Figure 2, the 4′−OH BDEs of pyranoanthocyanins 3′−radical and 3′,5′−di−radical are smaller than the 4′–OH BDEs in the gas phase of the parent pyranoanthocyanins. A similar trend is observed in the benzene phase. For instance, in Figure 2, the 4′–OH BDEs of the Pn-3-glc pyruvic acid 3′-radical, Pt-3-glc pyruvic acid 3′−radical, and Vitisin A 3′,5′−radical are 83.5, 78.6, and 77.4 kcal/mol, respectively. These values are significantly lower than the 4′–OH BDEs of the parent Pn-3-glc pyruvic acid (100.0 kcal/mol), Pt-3-glc pyruvic acid (92.8 kcal/mol), and Vitisin A (94.1 kcal/mol), respectively. Therefore, in the gas and benzene phases, the energy requirement for the hydrogen atom transfer reaction from 4′−OH is comparatively lower than the hydrogen atom loss process from 4′−OH in the parent molecules. This is primarily attributed to the formation of quinone in the singlet state, rather than the triplet state, which would otherwise promote the second hydrogen atom denaturation process.

While, after losing a proton from the 4′–OH in the B ring, the second PL reaction prefers proceeding on the A ring 7–OH group in the water phase. For instance, in Figure 4, the calculated 5′−OH PDE of the Pt-3-glc pyruvic acid 4′,12−di−anion is 52.6 kcal/mol, which is larger than that of 7–OH (38.6 kcal/mol). This result is principally ascribed to the fact that the three negative charges can be better delocalized on the 4′,7,12−tri−anion by conjugation over the entire skeleton, while the two negative charges may be concentrated on the B ring in 4′,5′,12−tri−anion, which would reduce the stability of the tri−anion.

## 5. Conclusions

In conclusion, we have applied DFT methods to study the multiple free radical scavenging reactions of anthocyanin derivatives called pyranoanthocyanins derived during wine storage and ageing. The molecular intrinsic cause for the antioxidant activities of pyranoanthocyanins as radical scavengers has been discussed in detail. The geometries of the pyranoanthocyanins were optimized first, and intramolecular hydrogen bonds between H2′ and O1, H4′ and O3′, and H6′ and O3 were detected in all the pyranoanthocyanins. The planarity of pyranoanthocyanins worsens with the increasing dielectric constant in the environment. Based on the optimized structures, the proton loss, electron transfer or hydrogen atom donation process were all considered for the three pyranoanthocyanins, and the intermediate species and the related reaction enthalpies were computed in different environments.

The environment influences the energy costs of various free radical-capturing mechanisms. By comparing the relative values of different reaction enthalpies, the thermodynamically preferred reaction pathway can be identified in different phases. The three pyranoanthocyanins can capture n_1_+1 free radicals. In the gas phase, all three pyranoanthocyanins scavenge free radicals through demethylation of the methoxy groups, followed by the HAT mechanism from the 4′−OH groups in the B ring (n_1_−De−HAT mechanism). In the benzene phase, all three pyranoanthocyanins release one proton from the hydroxyl group in the A ring or the carboxylic group in the D ring, forming pyranoanthocyanin anions. This follows the demethylation of the methoxy groups and the HAT mechanism from the 4′−OH groups in the B ring to trap free radicals (PL−n_1_−De−HAT mechanism). In the water phase, the three pyranoanthocyanins may dissociate as carboxylate anions. Pyranoanthocyanin carboxylates prefer performing multiple free radical-capturing reactions via the n_2_−PL−n_1_−De−ET mechanism, where n_2_ and n_1_ represent the number of phenolic groups and methoxy groups, respectively.

In the gas and benzene phases, after the loss of methyl groups from the methoxy unit, pyranoanthocyanins preferentially abstract the hydrogen atoms from the 4′–OH group, located in the guaiacyl group, similar to methoxy groups, resulting in the formation of quinone. In a water environment, after losing a proton from the 4′–OH in the B ring, the second proton loss reaction tends to occur on the 7–OH group in the A ring.

This work studies the multiple free radical scavenging mechanisms of pyranoanthocyanins based on theoretical calculations, providing a theoretical mechanistic explanation for the process of pyranoanthocyanins scavenging free radicals. However, more work is still needed to reveal the mechanism from experimental aspects. In future work, we will strive to experimentally validate our theoretical mechanisms.

## Figures and Tables

**Table 1:** Selected dihedral angles θ in degrees (◦).

Compound	Environment	*θ*(C6′–C1′–C2–C3)	*θ*(C6′–C1′–C2–O1)	*θ*(C4–C10–C9–C8)	*θ*(O1–C9–C10–C5)
Pn-3-glc pyruvic acid	gas	21.2	−158.4	−179.2	−179.7
	C6H6	22.4	−157.2	−179.6	179.9
	water	32.4	−147.8	−179.9	−179.9
Pn-3-glc pyruvic acid	gas	20.5	−159.9	−179.8	179.9
	C_6_H_6_	22.8	−157.6	−179.8	179.5
	water	26.1	−155.0	179.3	179.0
Vitisin A	gas	23.4	−156.9	−179.7	−180.0
	C_6_H_6_	26.7	−150.8	−179.6	−179.5
	water	32.5	−149.2	179.4	178.8
	Environment	*θ*(O1–C2–C3–C4)	*θ*(C10–C9–O1–C2)	*θ*(C10–C5–O13–C12)	*θ*(C10–C4–C11–C12)
Pn-3-glc pyruvic acid	gas	4.2	−2.7	−1.2	−3.9
	C6H6	3.3	−3.2	−1.6	−4.2
	water	2.4	−2.0	−1.1	−2.9
Pn-3-glc pyruvic acid	gas	2.5	−2.6	−1.4	−3.8
	C6H6	1.7	−3.0	−1.5	−4.1
	water	−0.2	−1.8	−0.6	−3.5
Vitisin A	gas	2.8	−2.7	−1.2	−3.7
	C6H6	1.6	−2.0	−1.5	−4.0
	water	−0.4	−1.8	−0.5	−3.4

## Data Availability

Data are contained within this article.
